# Cardiopulmonary involvement in Puumala hantavirus infection

**DOI:** 10.1186/1471-2334-13-501

**Published:** 2013-10-28

**Authors:** Johan Rasmuson, Per Lindqvist, Karen Sörensen, Magnus Hedström, Anders Blomberg, Clas Ahlm

**Affiliations:** 1 Department of Clinical Microbiology, Umeå University, SE - 901 85 Umeå, Sweden; 2 Department of Surgical and Perioperative Sciences, Umeå University, Umeå, Sweden; 3 Department of Radiation Sciences, Umeå University, Umeå, Sweden; 4 Department of Public Health and Clinical Medicine, Clinical Physiology, Heart Centre, Umeå University, Umeå, Sweden; 5 Department of Public Health and Clinical Medicine, Umeå University, Umeå, Sweden

**Keywords:** Haemorrhagic fever with renal syndrome, Hantavirus, Echocardiography, Computed tomography, Respiratory function tests, Natriuretic peptides

## Abstract

**Background:**

Hantavirus infections cause potentially life-threatening disease in humans world-wide. Infections with American hantaviruses may lead to hantavirus pulmonary syndrome characterised by severe cardiopulmonary distress with high mortality. Pulmonary involvement in European Puumala hantavirus (PUUV) infection has been reported, whereas knowledge of potential cardiac manifestations is limited. We aimed to comprehensively investigate cardiopulmonary involvement in patients with PUUV-infection.

**Methods:**

Twenty-seven hospitalised patients with PUUV-infection were examined with lung function tests, chest high-resolution CT (HRCT), echocardiography including speckle tracking strain rate analysis, ECG and measurements of cardiac biomarkers N-terminal pro-B-type natriuretic peptide (NT-ProBNP) and troponin T. Patients were re-evaluated after 3 months. Twenty-five age and sex-matched volunteers acted as controls for echocardiography data.

**Results:**

Two-thirds of the patients experienced respiratory symptoms as dry cough or dyspnoea. Gas diffusing capacity was impaired in most patients, significantly improving at follow-up but still subnormal in 38%. HRCT showed thoracic effusions or pulmonary oedema in 46% of the patients. Compared to controls, the main echocardiographic findings in patients during the acute phase were significantly higher pulmonary vascular resistance, higher systolic pulmonary artery pressure, lower left ventricular ejection fraction and impaired left atrial myocardial motion. Pathological ECG, atrial fibrillation or T-wave changes, was demonstrated in 26% of patients. NT-ProBNP concentrations were markedly increased and were inversely associated with gas diffusing capacity but positively correlated to pulmonary vascular resistance. Furthermore, patients experiencing impaired general condition at follow-up had significantly lower gas diffusing capacity and higher pulmonary vascular resistance, compared to those feeling fully recovered.

**Conclusions:**

In a majority of patients with PUUV-infection, both cardiac and pulmonary involvement was demonstrated with implications on patients’ recovery. The results demonstrate vascular leakage in the lungs that most likely is responsible for impaired gas diffusing capacity and increased pulmonary vascular resistance with secondary pulmonary hypertension and right heart distress. Interestingly, NT-ProBNP was markedly elevated even in the absence of overt ventricular heart failure. The method of simultaneous investigations of important cardiac and respiratory measurements improves the interpretation of the underlying pathophysiologic mechanisms.

## Background

Hantaviruses are rodent-borne RNA viruses that cause potentially lethal infections in humans worldwide [1]. The disease is generally transmitted by inhalation of viral particles shed in rodent excreta [2,3]. Typical clinical features of hantavirus infection include a capillary-leak syndrome with hypotension and oedema, together with coagulopathy and general symptoms such as high fever, headache and myalgia [1]. In Eurasia, hantavirus strains cause haemorrhagic fever with renal syndrome (HFRS), while hantaviruses in North and South America cause hantavirus pulmonary syndrome (HPS; also denominated hantavirus cardiopulmonary syndrome), leading to severe and often fatal heart and lung failure [1,4-6].

Infection with the European Puumala hantavirus (PUUV) causes a mild HFRS, characterised by coagulopathy and acute renal failure [7]. In addition, pulmonary involvement is very common in PUUV-infection, ranging from mild symptoms to severe lethal forms similar to HPS [8-12]. Previous studies of patients with HPS and PUUV-related HFRS have shown impaired lung function, pleural effusion and pulmonary oedema [4-6,9,13,14], along with an activated lower airway immune response reported in both syndromes [11,12,15-17]. Only few studies have addressed heart function in patients with hantavirus infection [5,9,18,19]. Electrocardiographic (ECG) abnormalities, including sinus bradycardia and T-wave changes, have been described in 35-57% of European patients with HFRS [18,19]. Echocardiographic data from PUUV-infected patients have mainly included information of morphological and visual descriptions of left ventricle function [9,19], however with few details of right heart function that more likely would be influenced as a consequence of PUUV-related manifestations within the lungs.

In the present study, we aimed to comprehensively study cardiopulmonary involvement in patients with PUUV-related HFRS using detailed state-of-the-art echocardiography including speckle tracking technique, ECG and cardiac biomarkers, together with high-resolution CT (HRCT) of the chest and lung function tests. We hypothesised that PUUV would target the lungs causing signs of pulmonary capillary leakage and impaired gas diffusing capacity, along with related changes in heart function.

## Methods

### Subjects

Twenty-seven hospitalised patients (18 women; median age 54 years, range 19–82 years) with serology-verified PUUV-infection admitted to the Department of Infectious Disease at the University Hospital, Umeå, Sweden, were included in a prospective study between January 2008 and March 2011. Within the same time-span another 20 patients (8 women) with PUUV-infection were hospitalised at our clinic, but were not included due to short hospitalisation (<2 days, n = 7), declined study participation (n = 8) or logistical reasons (i.e. public holidays, n = 5). Twenty-one patients were previously healthy. Out of the remaining six patients, one patient had well-regulated hypertensive heart failure, chronic obstructive pulmonary disease and a history of transient atrial fibrillation; one patient was treated for hypertension, had type-II diabetes and previous transient atrial fibrillation; one patient had type-II diabetes, rheumatic arthritis, Parkinson’s disease and was treated for hypertension; two patients were treated for hypertension and one patient received treatment for allergic asthma. Nineteen patients were non-smokers and eight were smokers, defined as current smoking or having smoked within the last 2 years.

Cardiopulmonary involvement was studied in the acute phase during hospitalisation and patients were re-evaluated at follow-up after 3 months. Clinical data was retrieved from the patients’ medical charts. One patient declined follow-up investigation. Twenty-five healthy age and sex-matched volunteers acted as control group for echocardiographic data. Echocardiographic data from a patient with a history of heart failure and pulmonary disease was omitted from comparison with controls. Furthermore, this patient did not undergo lung function tests or HRCT. The study was approved by the Regional Ethics Review Board at Umeå University. Participants were treated according to the Declaration of Helsinki and all gave written informed consent.

### Lung function

Lung function was evaluated using computerised Jaeger equipment (Würzburg, Germany) according to guidelines [20,21]. Vital capacity (VC), total lung capacity (TLC), forced expiratory volume in 1 second (FEV_1_), FEV_1_/VC and diffusing capacity of the lung for carbon monoxide (DLCO) using the single-breath method were recorded. Results were expressed as percentage of predicted value for each patient using European reference values [22,23].

### High-resolution computed tomography

HRCT studies of the lungs were performed with a 64-slice scanner (LightSpeed VCT, GE Healthcare, Milwaukee, WI, USA), without administration of intra-venous contrast. Images were obtained in one spiral series during full inspiration in supine position from lung apex to costo-phrenic angle and were then reconstructed into slice thickness 1.25 mm with 10 mm interval. Imaging parameters were 120 kV, mA min-max 150–700 and noise index 35. Reconstruction kernel for the thin slices was “bone plus” and window settings for viewing lung parenchyma were W1600/L-400 and for soft tissue W350/L50.

### Echocardiography

Echocardiographic examinations, measurements and analyses were performed by one examiner (P.L.), using a Vivid 7 echocardiograph (GE Medical Systems, Horten, Norway) equipped with an adult 1.5-4.3 MHz phased array transducer. Standard views from the parasternal long and short axis as well as apical four-chamber views were obtained. Blood flow velocities were acquired as proposed by American Society of Echocardiography [24]. All recordings were performed with a superimposed ECG. Off-line analysis was made using a commercially available software system (EchoPac version 8.0.1, GE Healthcare, Waukesha, WI, USA). Measurements of left atrial (LA) and ventricular (LV) dimensions including septal and posterior wall thickness were determined as recommended [25,26]. From the apical four-chamber view LV volumes at end-systole and end-diastole were measured and LV ejection fraction was estimated using Simpson’s bi-plane model [26]. Doppler recordings of LV filling measurements were made of trans-mitral early (E) and late diastolic (A) velocities calculating E/A ratio, together with E-wave deceleration time and isovolemic relaxation time (LV IVRT, expressed as ratio of the previous R-R interval from ECG), all used as a means to evaluate LV diastolic function [27]. From pulmonary venous flow, the systolic (S) and diastolic (D) flow velocities were measured and the S/D ratio was calculated, as additional aid to assess LV diastolic function [27]. Stroke volume was measured using stroke distance from LV outflow-tract systolic flow and cross-sectional area [24]. Cardiac output was calculated from stroke volume x heart rate. The peak gradient between right ventricle (RV) and right atrium was measured, using the simplified Bernoulli formula [24]. From that gradient, an estimated right atrial pressure of 7 mm Hg was added for all to calculate peak systolic pulmonary artery pressure (sPAP). Mean PAP was calculated from sPAP × 0.62 + 2 [28]. Pulmonary vascular resistance (PVR) was calculated from the equation mean PAP - estimated pulmonary capillary wedge pressure of 10 mmHg / cardiac output, as previously described [29]. From the same view, tricuspid annular plane systolic excursion (TAPSE) was determined, as an indirect measure of RV ejection fraction [30]. Pulmonary artery acceleration time was determined as a means to detect increased PVR [31]. From the RV free wall, RV IVRT was measured and indexed to the previous R-R interval [32]. Myocardial function measured both in LA and LV was assessed using speckle tracking echocardiography technique, from the apical four-chamber view as previously described [33,34]. Using this technique, measurements of mean strain rate in six segments of the LA and LV were performed. LA and LV strain rate values during ventricular systole, early diastole and late atrial diastole were then determined.

### Electrocardiography

Resting 12-lead ECG was recorded in the acute phase and at follow-up and was analysed by one examiner (M.H.).

### Measurements of cardiac biomarkers and routine laboratory analyses

N-terminal pro-B-type natriuretic peptide (NT-proBNP) and high-sensitivity cardiac troponin T (hs-cTnT) were analysed in plasma according to clinical routine at an accredited laboratory at the Department of Clinical Chemistry at Umeå University hospital. Samples were taken on day of inclusion, then every second day during hospitalisation and at follow-up. Similarly, C-reactive protein (CRP), lactate dehydrogenase, albumin, creatinine, D-dimer, fibrinogen and PK-INR were analysed, along with blood leukocyte and platelet counts.

### DIC-scoring

Patients were scored for presence of disseminated intravascular coagulation (DIC) according to templates based on the International Society on Thrombosis and Haemostasis criteria as described previously [35]. Overt DIC was indicated by a score of ≥5 points.

### Statistical analysis

SPSS (version 20.0; IBM, Armonk, NY, USA) was used for statistical analysis. As most variables included small sample numbers and thus not expected to be normally distributed, non-parametric tests were used. Continuous variables were expressed as median (25^th^-75^th^ percentiles) unless stated otherwise. Mann–Whitney U test was used for group comparisons and Wilcoxon signed-ranks test was used for paired within-group observations. Fisher’s exact test was used for comparisons of categorical data. Spearman’s correlation coefficient was used to test for correlations. All tests were two-sided and P-values < 0.05 were considered statistically significant.

## Results

### Clinical data

All 27 patients experienced symptoms typical for PUUV-infection and were hospitalised for 5 (4–7) days. Clinical findings and laboratory results are presented in Table 1. One-third of the patients were hypotensive (systolic blood pressure ≤ 90 mg Hg). Respiratory tract symptoms were present in 67%, most frequently expressed as mild to moderate dyspnoea or dry cough. Lowest pulse oximetry saturation when breathing ambient air was 94% (92–96) and 33% were given supplemental oxygen treatment. Patients requiring oxygen treatment had significantly higher creatinine levels and leukocyte counts, together with lower nadir albumin, when compared to those without need for oxygen (Table 2). Thrombocytopenia (platelet count <145 10^9^ L^-1^) and transient renal failure (indicated by creatinine >150 μmol L^-1^) were seen in 93% and 63% of the patients respectively. Compared to weight on admission, patients’ weight gain was 0.9 kg (0–2.4) during hospitalisation. No patient experienced chest pain, no one received dialysis and all survived. One patient required intensive-care treatment for pulmonary oedema and hypotension, receiving non-invasive mechanical ventilation and intra-venous fluid therapy. In another patient, the clinical course was complicated by pulmonary embolism diagnosed 19 days post onset of PUUV-infection and one patient developed a profusely bleeding duodenal ulcer 12 days after onset of disease.

**Table 1:** Clinical findings and laboratory results in acute phase and at follow-up in Puumala hantavirus infection

	**Acute phase**	**Follow-up**^**a**^
Number of patients	27	26
**Clinical findings**		
Hypotension (≤90 mmHg)	9 (33%)	0
Respiratory symptoms	18 (67%)	6 (23%)
Dyspnoea	14 (52%)	6 (23%)
Dry cough	10 (37%)	0
Oxygen treated	9 (33%)	0
Acute renal failure^b^	17 (63%)	0
Oliguria (<400 mL/24 hours)	3 (11%)	0
**Laboratory results**		
Leukocyte count max (3.5-8.8 10^9^/L)	8.8 (7.0-12.3)	6.4 (5.3-8.1)
CRP max (<3 mg/L)	115 (47–166)	<3 (<3- < 3)
Platelet count min (145–387 10^9^/L)	66 (51–90)	249 (208–304)
D-dimer max (<0.2 mg/L)	1.1 (0.7-1.7)	0.1 (0.1-0.2)
Creatinine max (<105 μmol/L)	177 (121–304)	69 (57–92)
Lactate dehydrogenase max (<3.4 μkat/L)	4.9 (4.3-6.4)	3.1 (2.8-3.5)
Albumin min (36–45 g/L)	28 (22–30)	45 (42–47)
*Cardiac biomarkers*		
NT-proBNP inclusion (<150 ng/L)	646 (222–1568)	-
NT-proBNP max (<150 ng/L)	1768 (585–5067)	62 (32–130)
hs-cTnT max (<15 ng/L)	7 (0–16)	0 (0–7)

^a^Laboratory parameters normalised at follow-up (P < 0.001 for all, Wilcoxon signed-ranks test), compared to the acute phase, ^b^ Defined as creatinine concentration >150 μmol L^-1^. CRP, C-reactive protein; hs-cTnT, high-sensitivity cardiac troponin T; max, maximum; min, minimum; NT-proBNP, N-terminal pro-B-type natriuretic peptide.

Clinical findings are presented as number of patients (%) with the respective finding, while laboratory parameters are expressed as median (25^th^-75^th^ percentiles). Reference values (decision level for heart failure or myocardial injury for NT-ProBNP and hs-cTnT respectively) are presented in parenthesis.

**Table 2:** Significant results in relation to need of oxygen and impaired general condition at follow-up

	**Oxygen treatment Yes / No**	**Impaired condition follow-up Yes / No**
**Laboratory results**		
Number of patients	9 / 18	13 / 13
Leukocyte count max (3.5-8.8 10^9^/L)	13.0 (9.1-15.7) / 7.8 (6.3-9.2)**	6.5 (5.6-7.9) / 5.4 (5.0-8.5)
Creatinine max (<105 μmol/L)	276 (186–352) / 143 (111–237)*	69 (59–92) / 68 (53–92)
Albumin min (36–45 g/L)	21 (19–27) / 29 (25–31)**	44 (41–49) / 45 (42–46)
*Cardiac biomarkers*		
NT-proBNP max (<150 ng/L)	8470 (2055–12511) / 860 (237–4024)**	75 (36–300) / 49 (15–108)
hs-cTnT max (<15 ng/L)	16 (8–22) / 6 (0–10)*	6 (0–8) / 0 (0–7)
**Echocardiographic results**		
Number of patients	9 / 17	11 / 10
Heart rate (beats/min)	90 (81–104) / 66 (58–84)*	70 (58–84) / 71 (65–79)
Left ventricle IVRT (%)	11.9 (9.4-15.4) / 9.5 (7.8-10.9)*	9.7 (8.6-11.4) / 10.2 (8.7-12.1)
Systolic pulmonary artery pressure, mmHg	47 (35–47) / 33 (26–39)**	34 (29–39) / 27 (27–32)
Pulmonary vascular resistance (WU)	3.7 (2.8-4.4) / 2.3 (1.8-3.1)*	3.1 (1.9-3.7) / 1.8 (1.5-1.8)*
Pulmonary artery acceleration time (ms)	90 (70–111) / 127 (103–147)*	123 (104–137) / 124 (117–144)
TAPSE (mm)	20 (17–24) / 24 (21–30)*	21 (18–23) / 26 (21–29)
**Lung function results**		
Number of patients	7 / 18	12 / 12
DLCO	53 (38–61) / 70 (62–82)*	74 (68–83) / 90 (84–101)*

DLCO, diffusing capacity of the lung for carbon monoxide; hs-cTnT, high-sensitivity cardiac troponin T; IVRT; isovolemic relaxation time (indexed to R-R interval); max, maximum; min, minimum; NT-proBNP, N-terminal pro-B-type natriuretic peptide; TAPSE, tricuspid annular plane systolic excursion; WU, Wood units.

Results are expressed as median (25^th^-75^th^ percentile). Laboratory reference values (decision level for heart failure or myocardial injury for NT-ProBNP and hs-cTnT respectively) are presented in parenthesis. P-values, determined by Mann–Whitney U test, are expressed as * < 0.05, ** < 0.01.

At follow-up (n = 26), patients were generally recovering. However, 13 patients (50%) reported impaired general condition, described as tiredness or fatigue, out of which six patients (23%) were still experiencing effort dyspnoea.

### Lung function

In the acute phase, 20 patients (80%) showed subnormal results (<80% of predicted value) for DLCO, while other parameters were within normal limits (Table 3). Patients requiring oxygen treatment had significantly lower DLCO, compared to patients not in need of oxygen (Table 2). There was no statistical difference in lung function when comparing female and male patients (data not shown).

**Table 3:** Lung function and chest high-resolution computed tomography results in acute phase and at follow-up

	**Acute phase**	**Follow-up**	**P-value**^**a**^
**Lung function results**			
Number of patients	25	24	-
Days post onset of disease	7 (5–9)	98 (88–115)	-
VC	103 (93–113)	112 (103–120)	<0.001
TLC	105 (92–115)	109 (95–112)	<0.05
FEV_1_	94 (77–104)	102 (93–117)	<0.001
FEV_1_/VC	94 (88–99)	95 (86–101)	ns
DLCO	67 (54–76)	83 (70–94)	<0.001
**Chest HRCT results**			
Number of patients	24	14^b^	-
Days post onset of disease	6 (5–7)	100 (94–110)	-
Pleural effusion	9 (38%)	0	-
Pulmonary oedema	5 (21%)	1 (4%)	-
Enlarged thoracic lymph nodes	4 (17%)	0	-
Pericardial effusion	2 (8%)	0	-
Pneumonic infiltrate	1 (4%)	0	-

^a^Determined by Wilcoxon signed-ranks test, ^b^Radiological follow-up (X-ray or HRCT, when indicated) were performed in 14 patients with pathological findings. DLCO, diffusing capacity of the lung for carbon monoxide; FEV_1_, forced expiratory volume in 1 second; HRCT, high-resolution computed tomography; TLC, total lung capacity; VC, vital capacity.

Results are expressed as median (25^th^-75^th^ percentile) as percentage of predicted value for lung function and number of patients (%) with the respective finding for chest HRCT.

Although DLCO had significantly improved at follow-up (Table 3), nine patients (38%) were still displaying subnormal DLCO. Furthermore, patients experiencing impaired general condition at follow-up had significantly lower DLCO, compared to those feeling fully recovered (Table 2).

### High-resolution computed tomography

**Figure 1:**
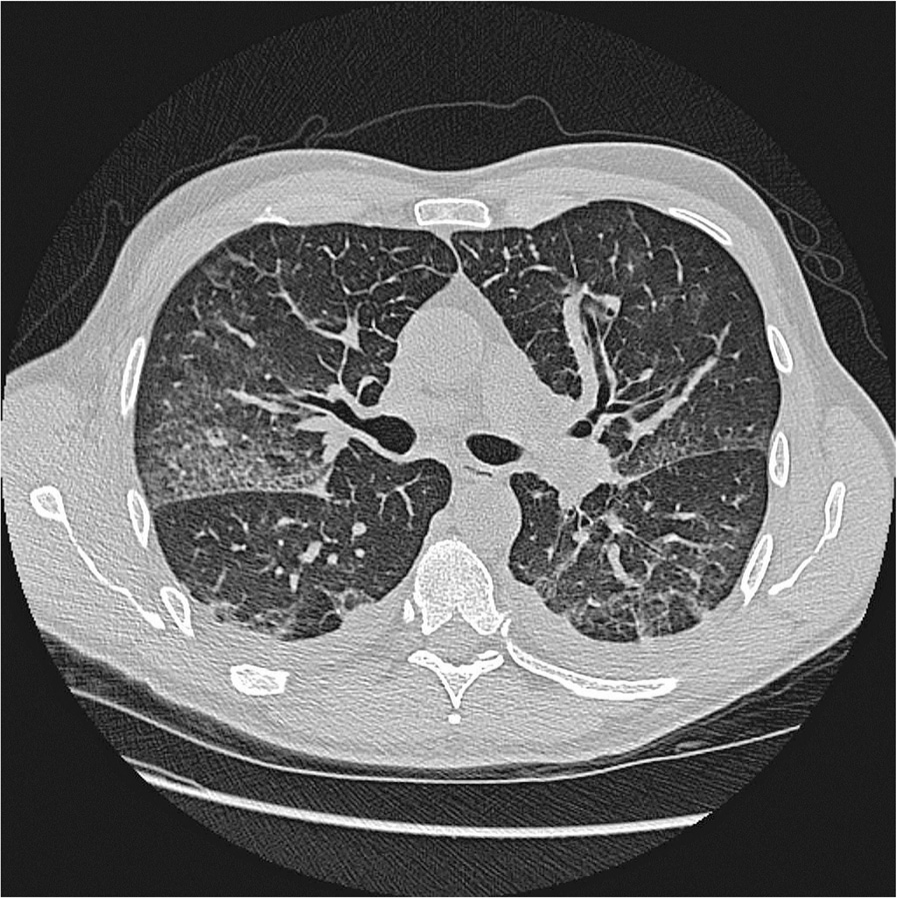
**Chest high-resolution computed tomography image from a patient with Puumala hantavirus infection.** Investigation was performed four days post onset of disease and shows bilateral pleural effusion and pulmonary oedema in a previously healthy non-smoking patient with pronounced clinical lung manifestations.

Abnormal chest HRCT was evident in 14 patients (58%). Thoracic effusions or pulmonary oedema (Figure 1) were found in 11 patients (46%). Enlarged thoracic lymph nodes and pneumonic infiltrates were also detected (Table 3). Patients with abnormal chest HRCT had lower DLCO (P < 0.05), higher CRP (P < 0.05), higher leukocyte count (P = 0.053) and were more likely to require oxygen treatment (P < 0.05), compared to patients with normal chest HRCT (data not shown). Patients with pulmonary oedema had higher leukocyte count (P < 0.05) and lactate dehydrogenase concentration (P < 0.05), lower nadir platelet count (P < 0.05) and a non-significantly lower DLCO (P = 0.064), compared to patients without pulmonary oedema (data not shown). There was no statistical difference in maximum creatinine concentrations between patients with abnormal HRCT or pulmonary oedema and those with normal findings (P = 0.241 and P = 0.197 respectively, data not shown).

Pathological HRCT findings were re-evaluated and normalised in all but one patient who 93 days after PUUV-infection had a progression of pulmonary oedema that was fully resolved when re-investigated after 240 days (Table 3).

### Echocardiography

Echocardiography findings showed that patients had significantly higher heart rate, lower LV ejection fraction, longer LV IVRT, larger systolic LV diameter and thinner LV posterior wall, when compared to controls (Table 4). Investigations of right heart function revealed that patients had significantly higher PVR and sPAP whereas pulmonary artery acceleration time was shorter, compared with controls. There was also a tendency to a longer RV IVRT in patients (P = 0.057, Table 4). Compared to controls, results from speckle tracking echocardiography revealed impaired atrial myocardial function in patients, indicated by significantly reduced strain rate during the late diastolic atrial contraction phase, measured both in LA and LV (Table 4). Compared to patients without need for oxygen supplementation, those requiring oxygen treatment had RV systolic involvement indicated by significantly higher PVR and sPAP, shorter pulmonary artery acceleration time and lower TAPSE, along with faster heart rate and longer LV IVRT (Table 2). There was also a tendency to a longer RV IVRT in patients requiring oxygen (P = 0.070). Patients with pulmonary oedema had lower cardiac output, compared to those without pulmonary oedema [3.7 (3.0-4.4) and 5.3 (4.4-6.1) respectively; P < 0.05]. There were no significant differences in any echocardiographic parameter, when comparing female and male patients (data not shown).

**Table 4:** Echocardiographic results in patients with Puumala hantavirus infection, compared to healthy controls

	**Healthy controls**	**Acute phase**	**Follow-up**
Number of patients	25	26^a^	21
Days post onset of disease	-	7 (6–9)	94 (89–114)
Age (years)	56 (48–65)	57 (47–65)	54 (45–64)
Females/Males	17 / 8	18 / 8	13 / 8
Heart rate (beats/min)	59 (52–67)	80 (60–91)**	70 (61–83)
**Left heart function**			
Left atrium diameter (mm)	35 (32–39)	35 (30–40)	36 (31–39)
Interventricular septum diastole (mm)	9 (8–11)	9 (8–11)	9 (8–10)
Left ventricular diameter diastole (mm)	49 (46–53)	49 (44–51)	47 (45–50)
Left ventricular diameter systole (mm)	30 (25–31)	31 (28–35)*	29 (28–31)
Posterior wall diastole (mm)	8 (7–9)	7 (7–8)*	7 (7–8)
Left ventricle ejection fraction (%)	0.70 (0.62-0.74)	0.60 (0.55-0.70)**	0.63 (0.59-0.66)
Left ventricle stroke volume (mL)	74 (61–86)	62 (53–80)	76 (69–85)‡
Cardiac output (L/min)	4.2 (3.6-5.0)	4.8 (4.0-5.7)	5.5 (4.4-6.4)
Mitral flow deceleration time (ms)	185 (148–225)	160 (136–188)	161 (124–209)
Mitral flow E/A	1.0 (0.8-1.3)	1.2 (1.0-1.6)	1.1 (0.8-1.4)
Pulmonary venous flow S/D	1.3 (1.0-1.5)	1.2 (1.0-1.9)	1.4 (1.2-2.0)
Left ventricle IVRT (%)	8.3 (6.6-9.8)	9.9 (8.9-11.9)*	10.0 (8.7-11.8)
**Right heart function**			
Systolic pulmonary artery pressure (mmHg)	26 (23–31)	36 (31–46)**	29 (27–36)
Pulmonary vascular resistance (WU)	1.7 (1.4-2.7)	2.9 (2.1-3.5)*	1.8 (1.6-3.1)
Pulmonary artery acceleration time (ms)	137 (129–145)	116 (90–142)*	124 (110–133)
Right ventricle IVRT (%)	4.8 (2.5-7.6)	7.3 (4.0-9.0)	5.0 (3.3-8.8)
TAPSE (mm)	23 (21–27)	24 (20–27)	22 (20–29)
**Left ventricular strain rate**			
Ventricular systole (1/s)	0.87 (0.76-0.96)	0.94 (0.77-1.10)	0.92 (0.75-1.13)
Early diastole (1/s)	0.98 (0.87-1.16)	1.00 (0.60-1.51)	0.89 (0.69-1.25)
Late atrial diastole (1/s)	0.90 (0.84-1.05)	0.70 (0.52-0.86)**	0.91 (0.60-1.12)‡‡
**Left atrial strain rate**			
Ventricular systole (1/s)	1.41 (1.06-1.69)	1.28 (0.93-1.69)	1.69 (1.37-1.99)
Early diastole (1/s)	1.40 (1.16-1.86)	1.24 (0.76-1.94)	1.71 (0.94-2.14)
Late atrial diastole (1/s)	1.70 (1.25-1.97)	1.34 (0.91-1.57)**	1.72 (1.26-2.13)‡

^a^One patient with heart failure was omitted from statistical comparison with controls. E/A, ratio of trans-mitral early to late diastolic flow velocities; IVRT, isovolemic relaxation time (expressed as ratio of R-R interval); S/D, ratio of pulmonary venous systolic to diastolic flow velocities; TAPSE, tricuspid annular plane systolic excursion; WU, Wood units.

Data are presented as median (25^th^-75^th^ percentiles). P-values, determined by Mann–Whitney U test or Wilcoxon signed-ranks test, are expressed as * < 0.05, ** < 0.01 comparing patients and controls; ‡ < 0.05, ‡‡ < 0.01 comparing patients in acute phase and at follow-up.

At follow-up, there were significant improvements in stroke volume and atrial myocardial contraction during late diastole (Table 4). Otherwise, all the abnormal findings for LV and RV function as well as pulmonary haemodynamic in the acute phase were returning to normal but had not changed significantly. Furthermore, patients experiencing impaired general condition at follow-up had significantly higher PVR, together with non-significantly higher sPAP (P = 0.091) and lower TAPSE (P = 0.052), when compared to those feeling fully recovered (Table 2).

### Electrocardiography

ECG was recorded in 27 patients in the acute phase 5 (5–7) days and in 22 patients at follow-up 114 (99–155) days post onset of disease. In the acute phase, pathological ECG was evident in seven patients (26%). T-wave changes were seen in all abnormal ECGs and atrial fibrillation was noted in two patients. There was no recorded ST-segment alteration or conduction disturbance. At follow-up, one patient still had atrial fibrillation, while the other had regained sinus rhythm, yet with remaining T-wave changes. The other patients’ ECGs were normal at follow-up.

### Cardiac biomarkers

NT-proBNP sampled on day of inclusion was pathologically elevated (>150 ng L^-1^) in 21 patients (78%), increased during hospitalisation and peaked in median 8 days post onset of disease (Table 1). The maximum level of NT-ProBNP was positively correlated to both PVR (r 0.538, P < 0.05) and sPAP (r 0.412, P = 0.071) but inversely correlated to DLCO (r −0.436, P < 0.05). Higher NT-proBNP concentrations were found in patients with pulmonary oedema (P < 0.01, data not shown). Eight patients (30%) displayed hs-cTnT concentrations above myocardial injury decision level (≥15 ng L^-1^). Patients with pathologically elevated hs-cTnT had higher NT-ProBNP levels, when compared to patients with normal hs-cTnT (P < 0.01, data not shown). Patients requiring oxygen treatment had significantly higher concentrations of NT-proBNP and hs-cTnT, compared to those not needing oxygen (Table 2). There were no significant differences in NT-ProBNP or hs-cTnT concentrations, when comparing male and female patients (data not shown).

At follow-up, NT-ProBNP and hs-cTnT concentrations had significantly returned to normal (Table 1), however there was an inverse correlation between patients’ NT-proBNP level and DLCO (r −0.576, P < 0.01).

### DIC-scoring

Overt DIC was established in 8 patients (30%). There were no significant differences in need of oxygen, DLCO or any parameter of cardiac function measured by echocardiography, when comparing patients with or without DIC (data not shown).

## Discussion

In the present study, we aimed to study cardiopulmonary involvement in patients with PUUV-infection. The results revealed that a majority of the patients experienced dry cough and/or dyspnea and almost all displayed impaired DLCO, indicating involvement of the lower airways and lung parenchyma. Even though DLCO improved after 3 months, it remained subnormal in more than one-third of the patients. Interestingly, patients reporting impaired general condition after 3 months had significantly lower DLCO compared to those who had recovered fully. This finding further implies pulmonary involvement in the pathogenesis of PUUV-infection and also indicates a possible explanation to the clinical observation of long-lasting fatigue commonly reported by the patients.

Thoracic effusions or pulmonary oedema, also found in patients with HPS [4-6], was demonstrated by HRCT in almost half of the patients. Echocardiography investigation did not reveal volume/pressure overload or evidence of elevated pulmonary capillary wedge pressure (normal LA/LV size and normal pulmonary venous and trans-mitral flow pattern). Therefore, we concluded that, similar to HPS, the pulmonary oedema was non-cardiogenic and instead related to increased capillary permeability that together with parenchymal inflammation most likely is responsible for the impaired gas diffusing capacity seen in the patients. The present HRCT results are supported by previous smaller CT or HRCT studies of patients with PUUV-infection [13,14].

As hypothesised, echocardiography results demonstrated abnormalities, mainly on the right-heart with increased pulmonary vascular resistance, leading to elevated pulmonary artery systolic pressure, also described in HPS and in patients with acute respiratory distress syndrome [5,36]. The pathogenesis of increased PVR is likely multi-factorial. Plausible mechanisms could include inflammation of pulmonary microvasculature, increased interstitial pressure, stress-induced arterial constriction and diffuse microembolism or thrombosis in the pulmonary vasculature. Hantavirus mainly infects vascular endothelial cells [37]. Both PUUV and HPS-related Sin Nombre virus have been demonstrated to replicate in pulmonary capillary endothelial cells, inducing inflammatory changes in the endothelium and capillary leakage that has been attributed to effects of vascular endothelial growth factor [11,16,17,37-40]. In addition, vascular endothelial growth factor has also been suggested to play a central role in development of pulmonary hypertension [41]. One may speculate that hantavirus infection in the pulmonary vascular endothelium leads to elevation of PVR and secondary pulmonary hypertension by means of endothelial inflammation and increased pulmonary interstitial pressure due to related vascular leakage. Pulmonary microembolism in PUUV-infection could be supported by the finding of pulmonary embolism in one patient in the current cohort and previously reported observations of symptomatic thromboembolism in survivors and evident scattered pulmonary microthrombosis in lethal cases [11,35]. In addition, DIC has been established in up to one out of four PUUV-infected patients [35,42]. However, in the present study, we could not establish any relation between the presence of DIC and increased PVR, making this mechanism less likely.

Similar to patients with bacterial sepsis [43], the PUUV-infected patients were hypotensive. This is likely a result of peripheral vasodilatation or myocardial depression due to pro-inflammatory cytokines [44,45], previously shown to be associated with hypotension in HFRS patients [46]. In contrast to the normal circulatory state of sepsis, PUUV-infected patients did not display an elevated cardiac output during the acute phase, when compared to controls or follow-up. This observation could speculatively be caused by myocardial depression in the patients, as described in HPS and in septic cardiomyopathy [43,47]. In the present study, patients displayed impaired atrial myocardial contraction. It is difficult to provide a clear explanation for this finding but it may be related to an increased LA wall stress and/or small increases in pulmonary capillary wedge pressure or to the higher heart rate in these patients. The lower LV ejection fraction and prolonged LV IVRT in PUUV-infected patients, compared to controls, were rarely subnormal and indicate trivial systolic and diastolic left ventricle dysfunction. Similar findings for LV ejection fraction have been reported in patients with PUUV-infection and patients surviving Sin Nombre virus-related HPS, whereas HPS fatal cases displayed pathologically reduced LV ejection fraction [5,19].

At follow up, compared to the acute phase, we found a significant increase in LV stroke volume and atrial myocardial contraction, while other determined echocardiography variables were non-significantly normalising, indicating slow functional haemodynamical recovery despite significant recovery in gas diffusing capacity. Notably, patients experiencing impaired general condition at follow-up had significantly higher PVR, which may suggest remaining PUUV-related pulmonary vascular effects in patients with prolonged clinical recovery.

The cardiac biomarker NT-ProBNP is used to detect heart failure but has not previously been evaluated in hantavirus infections. NT-ProBNP is secreted to the circulation as a consequence of myocardial stretch and dysfunction. It has vasodilatory and pro-diuretic effects that lead to reduced pre-load and lowered cardiac output [48,49]. The marked elevation of NT-ProBNP in the PUUV-infected patients is not easily explained, as overt left or right heart failure could not be established by echocardiography. However, it has been documented that NT-ProBNP can be increased in sepsis due to pro-inflammatory cytokines even without echocardiographic evidence of ventricular heart failure [50-53], as well as in patients suffering from tuberculosis and hepatitis C-infection [54,55]. Another contributing factor to increased NT-ProBNP in the PUUV-infected patients may be impaired renal elimination, since NT-ProBNP levels peaked during the late acute phase when patients were experiencing renal failure. However, the importance of renal clearance of NT-ProBNP is controversial and has been shown to account for only 15-20% of the total elimination [56,57]. Additionally, in almost all patients, an increase in NT-ProBNP was noted already on day of inclusion and prior to onset of renal failure. In the present study, we demonstrate that NT-ProBNP was inversely associated with patient’s gas diffusing capacity but positively correlated to PVR, in similarity with patients with pulmonary hypertension [58]. Furthermore, PUUV-infected patients with pulmonary oedema had higher levels of NT-ProBNP, possibly reflecting NT-ProBNP elevation in response to increased right ventricular load.

High-sensitivity troponin T, a biomarker of myocardial injury, was slightly elevated in one-third of the patients in the present study, indicating microscopic myocardial cell damage. Slightly less frequent than previously reported, pathological electrocardiograms were found in one-fourth of the PUUV-infected patients during the acute phase of disease [18,19]. No patient could be diagnosed with myocarditis, based on evaluation of clinical features, troponin T and ECG findings, which is in concordance with a previous study [19].

The major strengths of the present study are the simultaneous use of different diagnostic methods to evaluate cardiopulmonary manifestations and underlying pathophysiological mechanisms in a relatively large patient population during the acute and follow-up phases of PUUV-infection. However, there are some limitations. Many echocardiography variables are estimates. Although invasive monitoring using a pulmonary artery catheter would have given more accurate readings, in our opinion, this was not possible, given the presence of coagulopathy. Consequently, non-invasive measurements were chosen. Also, not all patients underwent follow-up echocardiography.

## Conclusions

Cardiopulmonary manifestations in PUUV-infection could be established in a majority of investigated patients, characterised by signs of pulmonary capillary leakage most likely responsible for the impairment of gas diffusing capacity and increased pulmonary vascular resistance with secondary pulmonary hypertension. In addition, levels of NT-proBNP were markedly elevated, indicating myocardial distress. Cardiopulmonary effects of PUUV-infection were long-lasting and had implications on patients’ recovery.

More studies are needed to further establish the mechanisms of increased pulmonary vascular resistance in PUUV-related hantavirus disease, and to evaluate the long-term effects of PUUV-infection on cardiopulmonary function.

## Abbreviations

DIC: Disseminated intravascular coagulation; DLCO: Diffusing capacity of the lung for carbon monoxide; HPS: Hantavirus pulmonary syndrome; HFRS: Haemorrhagic fever with renal syndrome; HRCT: High-resolution computed tomography; hs-cTnT: High-sensitivity cardiac troponin T; IVRT: Isovolemic relaxation time; LA: Left atrium; LV: Left ventricle; NT-ProBNP: N-terminal pro-B-type natriuretic peptide; PUUV: Puumala hantavirus; PVR: Pulmonary vascular resistance; RV: Right ventricle; sPAP: Systolic pulmonary artery pressure; TAPSE: Tricuspid annular plane systolic excursion.

## Competing interests

The authors declare that they have no competing interests.

## Authors’ contributions

JR conceived and designed the study, enrolled study subjects, collected data, performed statistical analyses and was principally responsible for manuscript preparation. PL contributed to study design, performed and analysed echocardiographic examinations and edited the manuscript. KS performed HRCT studies and interpretations and drafted the manuscript. MH contributed to study design, analysed ECG, gave assistance in interpretation of echocardiographic and cardiac biomarker results and drafted the manuscript. AB contributed to study design, assisted with interpretation of lung function and HRCT results and edited the manuscript. CA supervised study design, enrolled study subjects and edited the manuscript. All authors read and approved the final manuscript.

## Authors’ information

JR: MD, PhD student and consultant in infectious diseases with clinical interest in hantavirus infection and patient care. PL: PhD and associate professor with long experience in echocardiography and heart physiology. KS: MD and senior consultant in diagnostic radiology with special interest in pulmonary imaging. MH: MD, PhD student and senior consultant in cardiology. AB: MD, PhD, professor and senior consultant in respiratory medicine with long experience in pulmonary research and clinical care. CA: MD, PhD, professor and senior consultant in infectious diseases with a special interest in hantavirus research and clinical care.

## Pre-publication history

The pre-publication history for this paper can be accessed here:

http://www.biomedcentral.com/1471-2334/13/501/prepub
